# Improving newborn screening accuracy through genome sequencing, targeted metabolomics, and machine learning

**DOI:** 10.1186/s12920-025-02261-x

**Published:** 2025-11-19

**Authors:** Yuhan Xie, Gang Peng, Irina Tikhonova, Gregory Enns, Hongyu Zhao, Tina Cowan, Curt Scharfe

**Affiliations:** 1 Department of Genetics, Yale School of Medicine, New Haven, CT, USA; 2 Department of Biostatistics, Yale School of Public Health, New Haven, CT USA; 3 Department of Medical and Molecular Genetics, Indiana University School of Medicine, Indianapolis, IN USA; 4 Department of Pathology, Stanford University School of Medicine, Stanford, CA USA; 5 Department of Pediatrics, Stanford University School of Medicine, Stanford, CA USA

**Keywords:** Newborn screening, Next-generation sequencing, Metabolomics profiling, Machine learning, Dried blood spot, Metabolic disorder, Rare diseases, Molecular diagnostics

## Abstract

**Background:**

Newborn screening (NBS) enables early detection of metabolic disorders, but current tandem mass spectrometry (MS/MS) methods often lead to false positives and require confirmatory testing, causing diagnostic delays. We evaluated whether integrating genome sequencing, expanded metabolite profiling, and artificial intelligence/machine learning (AI/ML) could improve the accuracy of NBS.

**Methods:**

We analyzed dried blood spots (DBS) from 119 screen-positive cases identified by the California NBS program across four disorders: GA-I, PA/MMA, OTCD, and VLCADD. Genome sequencing was performed to identify variants in condition-related genes using ACMG guidelines, and an AI/ML classifier trained on previously generated metabolomic data was applied to differentiate true and false positives.

**Results:**

Genome sequencing confirmed 89% (31/35) of true positives based on the presence of two reportable variants. Among 84 false positives, 74% (62) had no variant, while 26% (22) carried a pathogenic/likely pathogenic variant or rare VUS in a condition-related gene. For VLCADD, half of false positives (15/29) were ACADVL variant carriers (*P* = 4.66 × 10⁻⁷). VLCADD biomarker levels were highest in patients, intermediate in carriers, and lowest in non-carriers, indicating that ACADVL variants elevate biomarker levels and increase false-positive rates. Metabolomics with AI/ML detected all true positives (100% sensitivity), while genome sequencing reduced false positives by 98.8%.

**Conclusion:**

Targeted metabolomics with AI/ML showed high sensitivity for identifying true positives, though its ability to reduce false positives varied by condition. Genome sequencing effectively reduced false positives but lacked sufficient sensitivity as a standalone test. The elevated false-positive rate among pathogenic variant carriers underscores the potential value of parental or prenatal carrier screening to improve NBS accuracy. Integrating genomic and metabolomic data may enhance NBS precision and enable earlier diagnosis and intervention for rare diseases.

**Supplementary Information:**

The online version contains supplementary material available at 10.1186/s12920-025-02261-x.

## Background

Inborn metabolic disorders (IMD) are a heterogeneous group of genetic conditions, with a collective incidence estimated to range from 1 in 800 to 5,000 live births worldwide [1–3]. Newborn screening (NBS) using tandem mass spectrometry (MS/MS) can detect over forty IMDs on the Recommended Uniform Screening Panel (RUSP) [4], enabling early intervention before life-threatening symptoms develop [5–8]. MS/MS-based dried blood spot screening identifies most affected infants and additional biochemical and/or DNA testing of all screen-positives is needed to confirm or exclude a final diagnosis. This two-tier strategy can result in repeated testing cycles, diagnostic delays, and unnecessary precautionary treatment for false-positive cases.

Incorporating next-generation sequencing (NGS) into NBS has the potential to streamline the diagnostic process after an abnormal screening result, and support NBS expansion to include new conditions [9, 10]. Several research studies have demonstrated feasibility for NGS from dried blood spots as a second-tier method to confirm metabolic cases identified in newborn screening [11–16]. The sensitivity of NGS in confirming known IMD cases ranged from 80% to 89%, highlighting that NGS alone lacks the performance needed to serve as a primary NBS method for most IMD’s on the RUSP [12, 14, 15, 17, 18]. Recently, genome sequencing has been proposed as a tool to expand NBS, particularly for conditions where biochemical markers cannot be identified through DBS testing [19–23]. However, incorporating sequencing into NBS for IMDs poses challenges due to genetic and metabolic variability across human populations [24–26], as well as difficulties in interpreting variants of uncertain significance (VUS) [27–29].

Previously, we showed that second-tier analysis of NBS data using AI/ML [30] and expanded metabolomic analysis [31] can reduce false positives for four conditions: glutaric acidemia type 1 (GA-I), propionic acidemia/methylmalonic acidemia (PA/MMA), ornithine transcarbamylase deficiency (OTCD), and very long-chain acyl-CoA dehydrogenase deficiency (VLCADD). In this study, we performed genome sequencing from dried blood spots (DBS) from 119 screen-positive cases (35 true positives, 84 false positives) reported by the California NBS program. For all 119 cases, we compared the newly generated sequencing results to: (1) the primary MS/MS screening data, (2) second-tier AI/ML analysis of that MS/MS data, and (3) previously generated targeted LC-MS/MS metabolomic data used to train a Random Forest classifier. Our results reveal important differences between genome sequencing and metabolomic profiling in resolving NBS results. Metabolomics with AI/ML accurately identified all true positives, whereas genome sequencing showed lower sensitivity, failing to detect pathogenic variants in some confirmed cases. The effectiveness of metabolomics in reducing false positives varied by disorder, while genome sequencing identified two reportable variants in only one false-positive case. Notably, a subset of false positives carried a single pathogenic variant in a gene associated with the screening result, suggesting that heterozygosity may underlie elevated analyte levels that trigger false-positive MS/MS results [12, 32–34]. While no single method provided a comprehensive solution, our integrative approach combining genome sequencing, metabolomic profiling, and AI/ML shows promise for improving the timely resolution of all screen-positive cases.

## Methods

### Study population

Residual DBS specimens and their corresponding NBS data were obtained from the California Department of Public Health (CDPH) for 119 infants born between 2005 and 2015 comprising 35 TP cases of GA-I (*n* = 6), MMA (*n* = 13), OTCD (*n* = 3), and VLCADD (*n* = 13), as well as 84 false-positive cases (15 GA-I, 31 PA/MMA, 9 OTCD, 29 VLCADD). All true positive cases labeled PA/MMA by the NBS program were ultimately confirmed to have MMA; no confirmed PA cases were included in the study. NBS data included 41 metabolic analytes measured by MS/MS and six clinical variables of birth weight (BW in grams), gestational age (GA in days), age at blood collection (AaBC in hours), infant sex, parent-reported race/ethnicity, and total parenteral nutrition (TPN) status.

### DNA extraction from DBS and sequencing

A single 3-mm punch was taken from a DBS sample using a PE Wallac instrument (Perkin Elmer, Santa Clara, CA, USA) and deposited into a 96-well plate. Three blank paper spots were punched between each sample to prevent cross contamination. DNA from DBS punches was isolated using the KingFisher Apex system with MagMax DNA Multi-Sample Ultra 2.0 kit (Fisher, Part#A36570) according to manufacturer protocol. Extracted DNA samples were quantified via Quant-iT dsDNA HS Assay kit (Invitrogen, cat# Q33232) on microplate reader (Molecular Devices SpectraMax M2). Fifty nanograms of genomic DNA was sheared to a mean fragment length of about 300 bp using focused acoustic energy (Covaris E220) and inspected using the Agilent TapeStation system. Following fragmentation, sequencing libraries were prepared with xGen™ cfDNA and FFPE DNA Library Prep MC kit (IDT, Part# 10006203) following the manufacturer protocol. The adapter-ligated DNA fragments were PCR-amplified using custom-made primers (IDT). During PCR, a unique 10 base index is inserted at both ends of each DNA fragment. Size of the final library construct was determined on Agilent TapeStation and quantification was performed by qPCR using the Kapa Library Quantification Kit (Roche, Part#KK4854). Samples were normalized to 2 nM and loaded onto an Illumina NovaSeq X Plus flow cell at a concentration optimized to achieve at least 160 Gbp of passing filter data per sample. Loading conditions were selected to maximize well occupancy and unique read output while minimizing duplicate reads associated with patterned flow cell technology. Sequencing was performed using 151 bp paired-end reads following Illumina protocols, with dual 10 bp indexes read in separate cycles. A 1% PhiX spike-in was included as a positive control in each lane to monitor run quality. Sequencing data were transferred in real time to the YCGA high-performance computing cluster. Sample-level coverage metrics, including mean coverage and the percentage of bases covered at ≥ 30×, 40×, and 50×, are shown in Supplementary Table 1.

### Sequence data analysis

Data processing of genome sequencing data included demultiplexing, reference genome alignment (GRCh37), variant calling using the GATK HaplotypeCaller for generation of gVCF files, and variant filtering using GATK3 [35]. Separate filtering criteria were applied for SNPs (DP < 4, QD < 2.0, FS > 60.0, MQ < 35.0, MQRankSum < -12.5, ReadPosRankSum < -8.0), indels (DP < 4, QD < 2.0,FS > 200.0, ReadPosRankSum < -20.0 and MQ < 35.0), and mixed variants (i.e., combination of SNPs and indels at a single position) (DP < 4, QD < 2.0, FS > 60.0 and MQ < 35.0). The final call set was created by combining all variants, followed by filtering to exonic regions. Annotation was then performed using ANNOVAR [36] and the Ensembl Variant Effect Predictor [37]. A custom script was employed to parse all variants identified in 16 genes associated with the studied metabolic conditions. These genes included *ABCD4*,* ACADVL*,* ACSF3*,* CUBN*,* GCDH*,* HCFC1*,* LMBRD1*,* MCEE*,* MMAA*,* MMAB*,* MMACHC*,* MMADHC*,* MMUT*,* OTC*,* PCCA*,* and PCCB*. In addition, filtering criteria were used to select all genomic variants based on population frequency thresholds (≤ 0.025 in gnomAD [38] and/or those classified as pathogenic or likely pathogenic (P/LP) in the ClinVar database [39]. Variant annotation and classification were performed based on ACMG standards and guidelines for the interpretation of sequence variants [40]. Screen-positive cases with two P/LP variants, one P/LP variant plus a VUS, or two VUSs were classified as true positives. Conversely, screen-negative cases with fewer than two P/LP variants or VUSs were considered successfully excluded as false positives. Additionally, to determine whether carriers of variants in the corresponding disease gene(s) were overrepresented among false positives, an enrichment analysis was performed. Fisher’s exact test was used to assess the statistical significance of this enrichment.

### Metabolomic data analysis

The 119 screen-positive cases were previously included in two studies: one that developed a Random Forest (RF) machine learning classifier using primary NBS data to improve prediction of true versus false positives [30], and another that validated an expanded metabolomics panel combined with RF for enhanced second-tier screening [31]. To summarize briefly, targeted metabolomic profiling was performed on single DBS punches using a rapid, high-throughput LC-MS/MS method developed for second-tier NBS. This targeted approach tests 121 analytes, including all primary NBS markers, disease-associated metabolites identified through untargeted discovery, and newly detected isobaric species (see Table 1 in [31]). Chromatographic separation was performed using a 3-minute run with multiple reaction monitoring (MRM), allowing resolution of key isobaric compounds, particularly among long-chain acylcarnitine and hydroxy-acylcarnitine species. Internal standards were included in the analyses, and all samples were processed and analyzed together to avoid batch effects. Quality control was confirmed through tight clustering of technical replicates in principal component analysis. To classify true versus false positives, a RF model was trained using either metabolite peak areas or response ratios as input features. Model performance was evaluated using repeated leave-one-out cross-validation, with sensitivity and area under the curve (AUC) calculated for each disorder. Full details of the targeted metabolomics method and classifier development are available in [31].

**Table 1:** Performance of second-tier methods in screen-positive classification

NBS 1st -Tier			AI/ML (2nd tier)			Metabolomics + AI/ML (2nd tier)			Genome sequencing (2nd tier)		
Disease	TP	FP	FP Elim	% FP Red	PPV	FP Elim	% FP Red	PPV	FP Elim	% FP Red	PPV
GA-1	6	15	15	100%	100%	12	80%	67%	15	100%	100%
PA/MMA	13	31	17	55%	48%	24	77%	65%	31	100%	100%
OTC	3	9	9	100%	100%	9	100%	100%	9	100%	100%
VLCADD	13	29	0	0%	31%	12	41%	43%	28	97%	93%

*TP* true positives, *FP* false positives, *FP Elim.* number of false positives eliminated, *%FP Red* % of false positives reduced

In this study, we leveraged the 119 cases to conduct a four-way comparison of RF classification outcomes, expanded metabolomics, and genome sequencing results against the ground-truth labels (i.e., true or false positive) assigned by the California NBS program (Supplementary Table 1). We used prediction results reported by Peng et al. [30], based on a dataset of 2,777 samples from the California NBS program, including 235 true positives for GA-I, PA/MMA, OTCD, or VLCADD, and 2,542 false positives who initially screened positive but were later confirmed unaffected. Predictions were generated using a RF model with 10-fold cross-validation and 1,000 replications. Since the original study did not assign a single classification label per sample (i.e., predicted true positive or false positive), we applied a post hoc labeling approach. Using the same cutoff threshold as Peng et al. [30]—selected to preserve the original model’s sensitivity—we classified each sample in all 1,000 replications and assigned a final label based on the majority vote across replications. To replicate the metabolomics study by Mak et al. [31], we trained a Random Forest (RF) model on 186 samples (36 true positives, 150 false positives) to distinguish true from false positives. Model performance was evaluated using leave-one-out cross-validation (LOOCV), ensuring each sample was independently assessed. The model achieved 100% sensitivity, correctly classifying all true positives. To assess robustness, the LOOCV procedure was repeated 20 times. To compare metabolite marker values across groups—including true positives, false positives with or without relevant gene variants, and those with variants in unrelated genes—we used the Wilcoxon test to calculate *p*-values. To visualize overlap in correctly classified samples across methods, UpSet plots were generated for both true and false positives. Statistical analyses, graphs and design of the research was done in R software 4.4.2 [41] using these R packages: randomForest [42], ggplot2 [43], ggsignif [44], ggpubr [45], and ComplexUpset [46, 47].

## Results

### Genome sequencing identifies reportable variants in screen-positive cases

**Fig. 1:**
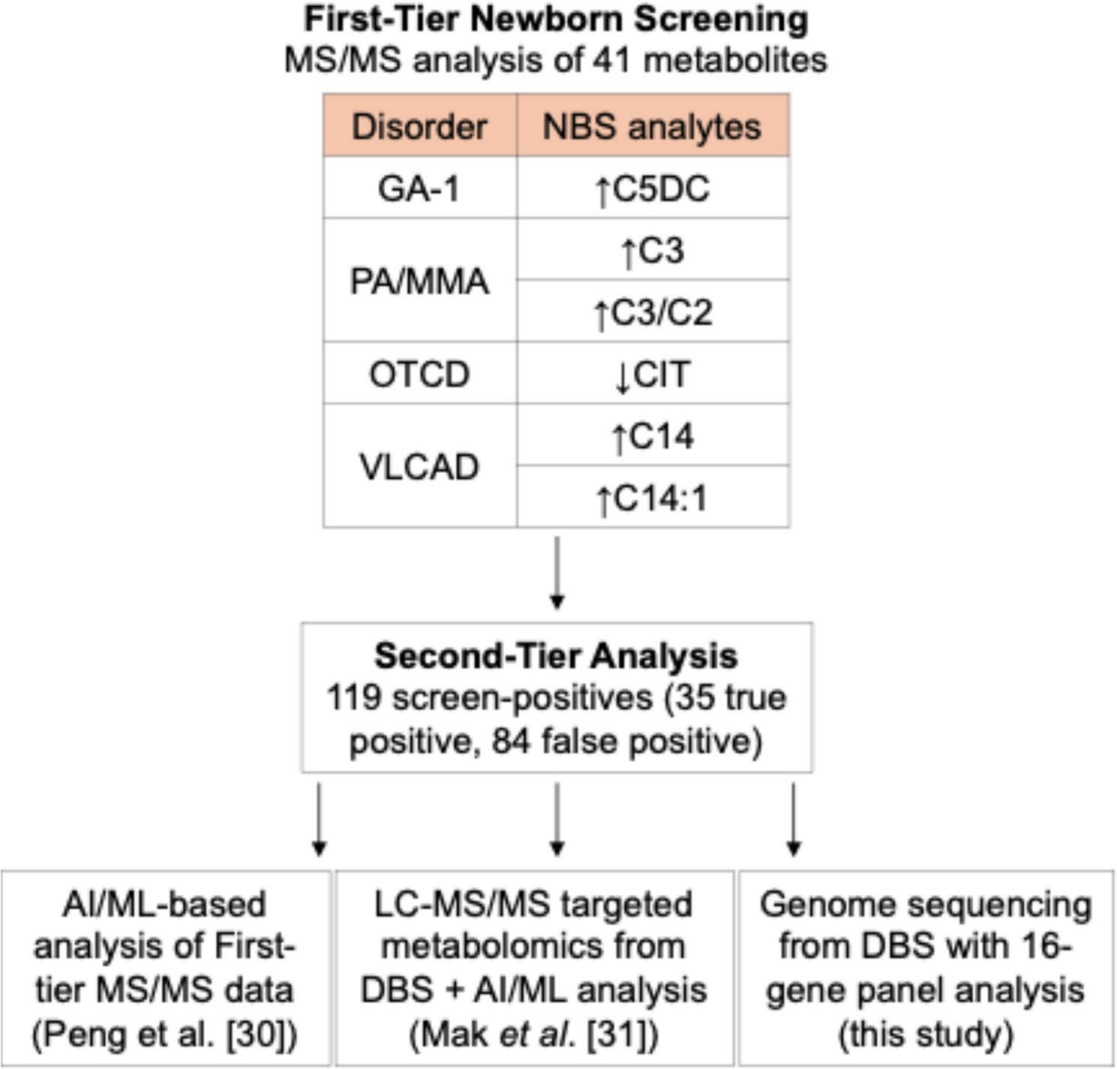
Testing flow for newborns with positive NBS results. Shown are the analysis steps for 119 newborns who screened positive by first-tier MS/MS in the California NBS program. Based on follow-up diagnostic data, 35 cases were classified as true positives and 84 as false positives. We performed genome sequencing on all samples and compared the results to: (1) the original first-tier MS/MS screening data, and to previously generated datasets for these same samples, including (2) AI/ML-based analysis of the MS/MS data [30], and (3) targeted LC-MS/MS metabolomic data analyzed using a Random Forest classifier [31]

Genome sequencing was performed on 119 screen-positive cases, comprising 35 true positives and 84 false positives (Fig. 1). Among the 35 true-positives, 31 (88.6%) had a reportable finding consistent with metabolic screening results. Of these, 23 carried two P/LP variants, including six with homozygous P/LP and three with two P/LP detected in trans (Fig S1A-C). Three cases had a hemizygous P/LP sequence change in the OTC gene, one of which involved a 1.89 Mb deletion at chromosome Xp11.4 (Fig S2). Additionally, four cases had one P/LP and one VUS, while one case carried two VUS in *ACADVL* associated with their NBS results. The remaining four patients had only a single heterozygous variant in a gene associated with their NBS results. This included two cases with a P/LP variant in *ACADVL*, one with a P/LP in *MMACHC*,* and* one with a VUS in *MMAA*.

Among the 84 false positive cases, one individual had two reportable *ACADVL* variants (p.Trp249Ser and p.Arg538Trp), aligning with their VLCADD-positive screening results. The remaining 83 cases either had no detected variant (*n* = 62) or a single heterozygous variant (*n* = 21) in a gene associated with their NBS findings. Among these 21 cases, 15 carried a known P/LP variant, while 6 had a rare VUS (*N* = 5) in a gene corresponding to the NBS result. Notably, among the 29 VLCADD false positives, 15 carried a heterozygous P/LP variant or VUS in *ACADVL*, while 14 had no such variant. A pairwise comparison between the groups revealed a significant association between VLCADD false-positive cases and carriers of a rare ACADVL variant (Fisher’s exact test, *P* = 4.66 × 10⁻⁷). In contrast, no significant enrichment of PA/MMA gene variant carriers was found in PA/MMA false positives (*P* = 0.314), with 6 of 31 MMA false positives carrying a P/LP variant or VUS in an MMA-associated gene consistent with their NBS result. Additionally, no enrichment of variant carriers was observed in GA-I and OTCD screen-positive cases, as no carriers were identified in these two diseases.

### Metabolite marker level differences among screen-positive groups

**Fig. 2:**
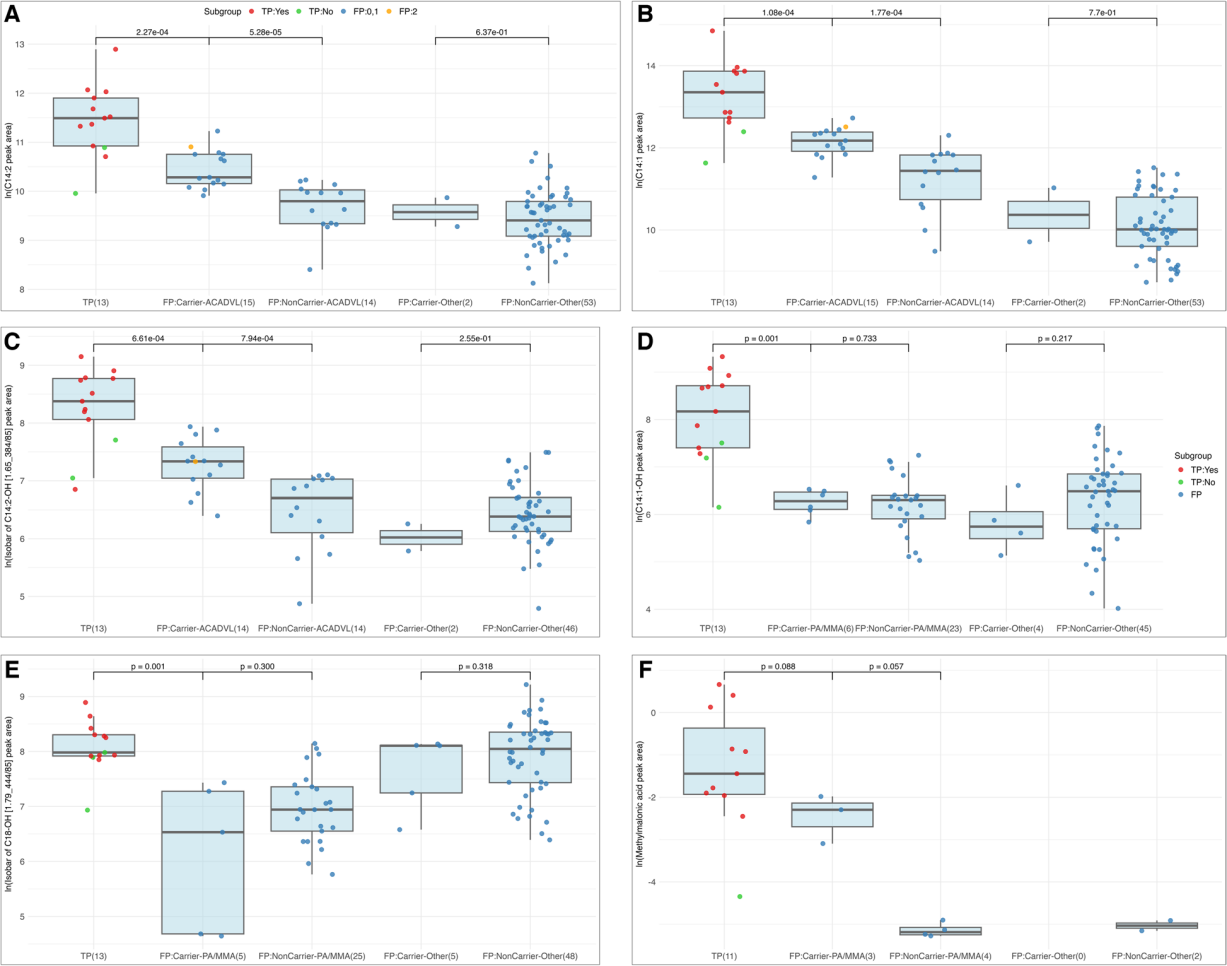
Metabolite marker profiles differentiate true- and false-positive cases. Boxplots show the distribution of natural logarithm (ln)-transformed metabolite peak areas across five groups of screen positives for VLCADD (**A-C**) and PA/MMA (**D-F**). From left to right, groups include: true positives, false positives with a rare variant (P/LP or VUS) in the associated gene (carriers), false positives without such variants (non-carriers), and two groups of false positives for other conditions (carriers and non-carriers). Newborns are color-coded as follows: true positives with two variants in ACADVL (**A–C**) or a PA/MMA-associated gene (**D–F**) in red; true positives with only one or no variants in green; and false positives in blue. *P*-values (Wilcoxon tests) indicate pairwise comparisons between groups, with sample sizes in parentheses. Numbers on the x-axis represent samples with available metabolite values, while those with missing values for the respective marker were excluded from analysis. Metabolite levels were highest in true positives, followed by false positives with a variant, and lowest in non-carriers

We previously identified metabolite markers important for true- and false-positive classification in second-tier newborn screening [31]. Here we selected the top-3 metabolite features identified for VLCADD (C14:2, C14:1, and an isobar of C14:2-OH [designated 1.65_384/85 ]) and for PA/MMA (C14:1-OH, an isobar of C18-OH [1.79_444/85], and methylmalonic acid), and compared their levels across screen-positive groups in relation to our sequencing results. We analyzed metabolite level differences across five groups: VLCADD true positive patients (*n* = 13), two VLCADD false positive groups including *ACADVL* variant carriers (*n* = 15) and non-carriers (*n* = 14), and two VLCADD screen negative groups including *ACADVL* variant carriers (*n* = 2) and non-carriers (*n* = 53). Pairwise comparisons revealed that metabolite levels were highest in VLCADD patients, followed by VLCADD false positives with an *ACADVL* variant and then *ACADVL* non-carriers (Fig. 2A-C). Notably, metabolite levels in VLCADD false positive *ACADVL* non-carriers closely resembled those of VLCADD screen negative individuals. In comparison for PA/MMA screen-positives (Fig. 2D-F), metabolite levels were higher in PA/MMA true positive cases compared to PA/MMA false positives. While screen-positive individuals with an PA/MMA gene variant tended to have higher metabolite levels, these differences were not statistically significant.

### Assessing sequencing, metabolomic and AI-driven analysis in resolving screen-positives

**Fig. 3:**
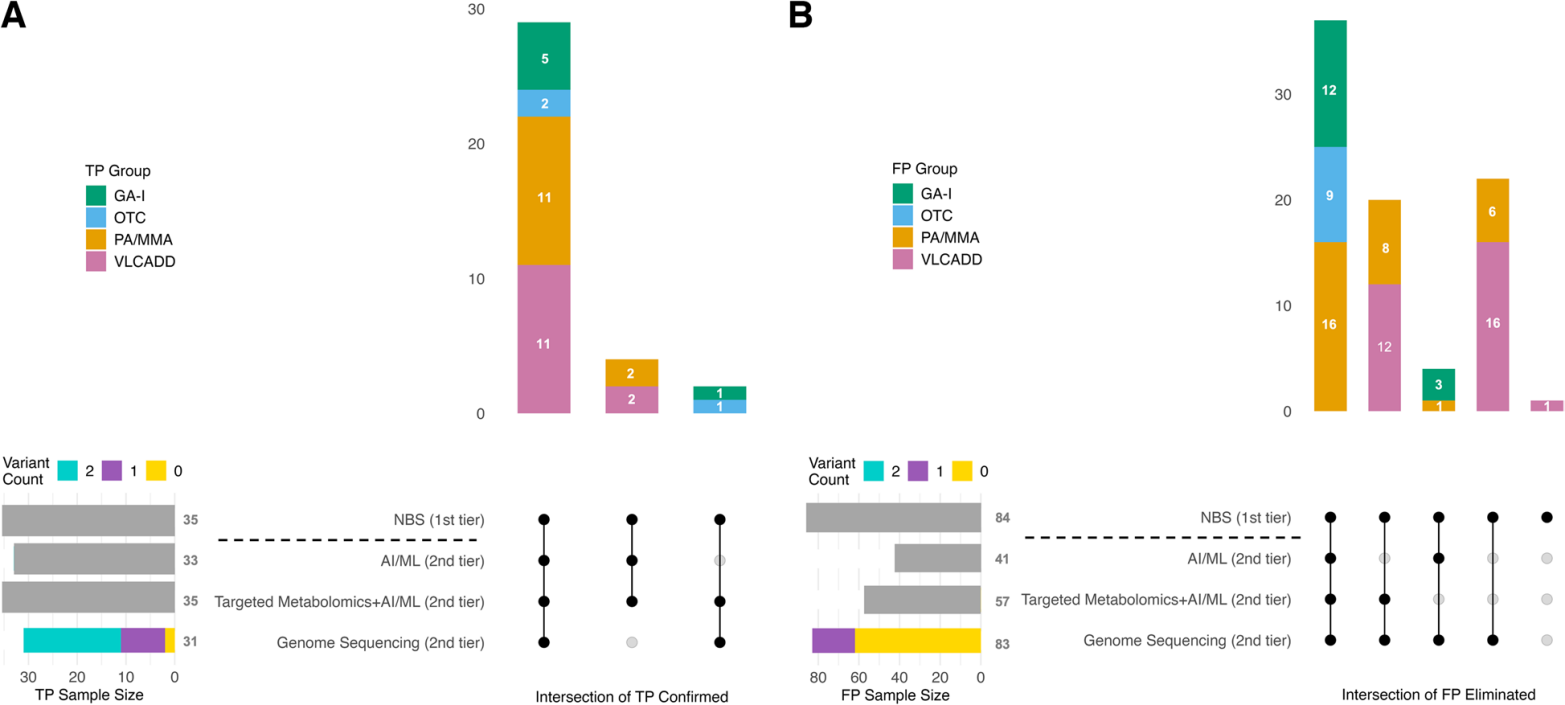
Resolution of screen-positive cases using genome sequencing, metabolomics, and AI/ML-enhanced analysis. UpSet plots illustrate the overlap among three second-tier methods—AI/ML-enhanced MS/MS data analysis, targeted metabolomic profiling, and genome sequencing—in resolving NBS-confirmed true positives (**A**) and false positives (**B**). Horizontal bars on the left show the total number of screen-positive cases identified by each method. For genome sequencing, color-coded segments indicate the number of variants (0, 1, or 2) detected among all screen-positive cases. Bars for the other methods are shown in grey, as variant information is not applicable. Vertical bars represent the number of cases identified by each specific combination of methods and are color-coded by disease. Connected dots below the vertical bars indicate which methods contributed to each combination

We previously studied the 119 screen-positive cases using AI/ML-enhanced analysis of NBS data [30] and metabolomics profiling from DBS [31] to improve second-tier screening (Fig. 1). Among the 35 true positives, two cases (1 GA-I, 1 OTCD) were misclassified as false positives by our AI/ML-based analysis, as their metabolic marker concentrations were below established disease-specific cutoff values. As a result, they were not initially flagged as screen-positive and were later identified through other means within the NBS program. While genome sequencing from DBS confirmed these two cases, it failed to detect the causative variants in four true positives, including two with PA/MMA and two with VLCADD. However, these cases were correctly identified by both AI/ML-based data mining and metabolomics profiling. In contrast, our second-tier metabolomics-AI/ML-enhanced analysis based on a 100% classification sensitivity (i.e., correctly classify all true positives) identified all 35 true positives (Fig. 3A).

Among the 84 screen-positives later confirmed as false positives by the NBS program, our AI/ML-based analysis of primary MS/MS data correctly identified 41 (48.8%) as false positives (Fig. 3B). In comparison, our metabolomics-AI/ML-enhanced analysis missed 4 of these 41 false positives but correctly identified an additional 20, resulting in a false positive detection sensitivity of 67.9% (57 out of 84). Notably, 27.3% (23 of 84) of the false positives reported by the NBS program were misclassified as true positives by both our AI/ML-based analysis and metabolomics profiling. Among the 23 false positives, nearly half (*n* = 11, 47.8%) carried a single heterozygous P/LP variant or a rare VUS in a metabolic disease gene corresponding to their NBS result, including 9 cases of VLCADD and 2 cases of MMA. One false positive carried two reportable *ACADVL* variants, which prevented a definitive classification as a false positive by any of the three methods used.

The performance of the three methods applied to second-tier screening varied across disorders (Table 1). AI/ML-based analysis of NBS data achieved a sensitivity of 94.3% for confirming true positives (33 of 35), while reducing false positives by 100% for GA-I and OTCD, 54.8% for PA/MMA, and 0% for VLCADD. Metabolomics-AI/ML-enhanced analysis based on a 100% classification sensitivity, reduced false positives by 80% for GA-I, 77.4% for PA/MMA, 100% for OTCD, and 41.4% for VLCADD. Genome sequencing showed a sensitivity of 88.6%, eliminating all false positives for GA-I, PA/MMA, and OTCD, and reducing false positives by 96.6% for VLCADD.

## Discussion

Tandem mass spectrometry-based newborn screening (MS/MS-NBS) is highly effective in detecting most affected infants. However, it often requires additional testing, which can lead to diagnostic delays and unnecessary treatment of false positive cases. Genome sequencing offers a promising method to enhance diagnostic accuracy and broaden NBS capabilities, yet its limitations prevent it from being a standalone method. Prior studies have reported a sensitivity of 80–89% for confirming inborn errors of metabolism, highlighting the need for complementary approaches to improve screening outcomes.

This proof-of-concept study investigates the integration of genome sequencing, metabolomics profiling, and AI/ML-enhanced analysis to enhance the sensitivity and specificity of NBS. To simulate second-tier testing, we analyzed dried blood spots from 119 screen-positive cases, assessing each method’s ability to differentiate true positives from false positives across four disorders: GA-I, PA/MMA, VLCADD, and OTCD (Fig. 1). By aiming for 100% sensitivity in detecting true positives, our integrated approach reduced false positives by 98.8%, showing significant potential to refine NBS and improve diagnostic accuracy (Table 1). This performance was primarily driven by the effectiveness of metabolomics profiling combined with AI/ML in confirming true positives, while genome sequencing played a crucial role in reducing false positives (Fig. 3). Notably, sequencing identified two reportable DNA variants in only one false-positive case, whereas all other false positives had either no variant or only a single variant in a gene associated with the NBS result. This additional genetic information allowed for the identification of 27.4% (*n* = 23) of false positives that metabolomics alone could not resolve, further enhancing diagnostic precision.

Nevertheless, this approach has inherent limitations. Genome sequencing failed to detect two pathogenic variants in approximately 11.4% of true positive cases, highlighting its potential for false-negative results. Additionally, cases with only one or no identified variants cannot be readily ruled out as false positives, particularly when their metabolic profile closely approaches the threshold for true positive cases. Indeed, we found that false positive variant carriers exhibited distinct metabolic profiles that differentiated them from both true positives and false positive non-carriers (Fig. 2). This suggests that incorporating metabolic differences (e.g., relative to the standard deviation of true positive profiles) along with genetic findings (e.g., identifying only a single variant) could improve false positive classification.

An additional limitation is that the targeted metabolomics data were obtained from stored DBS specimens, some of which had been archived for extended periods. Prior work by Mak et al. [31] noted that certain known metabolites—such as 3-hydroxyglutaric acid, homocystine, and methionine—were not reliably detected in older samples, likely due to degradation over time. Although we prioritized a robust panel of 121 analytes and conducted LC–MS/MS analysis under controlled conditions, future validation with fresh specimens will be important to confirm the stability and diagnostic utility of individual metabolites. This will also support broader clinical implementation and inter-laboratory standardization.

Separately, we observed that several long-chain acylcarnitine isobars (C14:1OH and C18OH) emerged as top features in the machine learning classification of PA/MMA cases (Fig. 1). While these are not traditional biomarkers for PA/MMA, their consistent selection by the classifier suggests they may reflect broader metabolic disturbances or subtype-specific patterns. Further investigation into their biological relevance may help elucidate the metabolic diversity underlying PA/MMA and refine future marker panels.

Further complexities emerged in the assessment of VLCADD cases. Two true positive cases, each carrying a single P/LP variant in *ACADVL*, exhibited metabolic marker levels that closely resembled those of false positive *ACADVL* carriers. This finding underscores the significant diagnostic challenge of distinguishing true positive VLCADD cases from false positive variant carriers. Notably, these patterns were not consistent across all disorders. While VLCADD exhibited characteristic metabolic profiles among false positive carriers, such profiles were absent for OTCD and GA-I. Additionally, the metabolic profiles of false positives in PA/MMA lacked distinctiveness, likely due to the heterogeneity of PA/MMA, which encompasses multiple genetic subtypes, including mut+/−, cobalamin disorders, and other subgroups. The absence of confirmed PA cases in the true positive cohort is a limitation, as it remains unknown how well the current approach performs in detecting true PA cases.

To address this, we sequenced an additional set of 46 false positive cases (Supplementary Table 2) to assess whether increasing the sample size would enhance statistical power for detecting enrichment of heterozygous variant carriers among VLCADD and PA/MMA false positives. While enrichment of heterozygous variant carriers in PA/MMA was non-significant (*P* = 0.314) in the initial 84 false positive set, it became significant when combining the 84 false positives with the additional 46 (*P* = 0.0455). Furthermore, in the combined VLCADD set, statistical power increased further (from *P* = 4.66 × 10⁻⁷ to *P* = 4.33 × 10⁻¹¹). Moving forward, a key goal in NBS research will be to define phenotypic subgroups among PA/MMA screen-positives, which will require expanding the sample size for each group. Establishing distinct metabolic profiles for phenotypic subgroups in PA/MMA and other IMD’s could enable more precise classification by integrating both metabolic and genetic data, ultimately improving diagnostic accuracy.

Expanding newborn screening beyond traditional IMDs offers a promising path for earlier detection and intervention across a wider range of conditions. We hypothesize that analysis of newborn metabolic profiles—whether from first-tier MS/MS or second-tier targeted metabolomics—can support interpretation of sequence variants in disorders beyond classical metabolic diseases. This approach assumes that genetic changes outside core metabolic pathways may still disrupt fundamental metabolic processes, leading to detectable shifts in metabolites such as amino acids and acylcarnitines. Emerging research supports this hypothesis. For instance, in Duchenne Muscular Dystrophy (DMD), a candidate disorder for NBS expansion [48, 49], studies have identified significant disruptions in amino acid, energy, and lipid metabolism, which correlate with the disease’s pathological features [50]. These findings suggest that metabolic profiling could serve as a valuable tool in DMD screening and diagnostics. Similarly, metabolomic analyses in cystic fibrosis (CF) have identified distinct metabolic signatures. A study of the sweat metabolome in screen-positive CF infants revealed novel disease-associated metabolites, highlighting the potential of metabolomic profiling to uncover unique CF biomarkers [51]. In addition, our findings may inform both NBS and prenatal genetic carrier screening, as many parents are offered testing for rare conditions included in NBS panels [52]. Identifying carrier status prenatally could help explain false-positive NBS results and improve screening accuracy. By establishing robust disorder-specific metabolic profiles, we could enhance screening precision, reduce false positives, and support earlier, more accurate diagnoses—ultimately improving health outcomes for affected children.

## Conclusion

Integrating genome sequencing, metabolomic profiling, and AI/ML significantly improves NBS accuracy by enhancing sensitivity and reducing false positives, though effectiveness varies by disorder. Metabolomics in combination with AI/ML reliably identified true positives, while sequencing helped reduce false positives but lacked sufficient sensitivity alone. Our findings suggest that pathogenic variant carriers are predisposed to false positives, highlighting the potential value of prenatal carrier screening in refining NBS outcomes. Integrating genome sequencing and metabolomic profiling into NBS holds the potential to expand the scope of detectable disorders beyond traditional IMDs. This approach could lead to earlier diagnoses and interventions for a variety of rare diseases, thereby enhancing patient care and management.

## Supplementary Information

Supplementary Material 1.

Supplementary Material 2.

## Data Availability

The de-identified residual dried blood spot (DBS) specimens and MS/MS screening data from the California Biobank used in this project (SIS request number 886) were obtained with a waiver of consent from the Committee for the Protection of Human Subjects of the State of California (project no. 13-05-1236) and in accordance with the CDPH Biospecimen/Data Use and Confidentiality Agreement. Under California law, these specimens and all data derived from the newborn screening program are confidential and subject to strict administrative, physical, and technical safeguards. Researchers are prohibited from sharing biospecimens or depositing individual-level data derived from these specimens in public repositories. Investigators seeking access to comparable materials or data must submit a separate application to the California Department of Public Health. Summary-level data supporting Figs. 1, 2 and 3 are provided in Supplementary Table 1.

## Abbreviations

NBS: Newborn screening
DBS: Dried blood spots
MS/MS: Tandem mass spectrometry
AI/ML: Artificial intelligence/machine learning
GA-I: Glutaric aciduria type 1
PA/MMA: Propionic acidemia/Methylmalonic acidemia
OTCD: Ornithine transcarbamylase deficiency
VLCADD: Very long-chain acyl-CoA dehydrogenase deficiency
P/LP: Pathogenic/likely pathogenic
VUS: Variant of uncertain significance
IMD: Inborn metabolic disorders
RUSP: Recommended Universal Screening Panel
NGS: Next-generation sequencing
CDPH: California Department of Public Health
BW: Birth weight
GA: Gestational age
AaBC: Age at blood collection
TPN: Total parenteral nutrition
RF: Random Forest
DMD: Duchenne Muscular Dystrophy
CF: Cystic fibrosis
CPS: Cancer predisposition syndromes
