# Supplementary material for: Improving newborn screening accuracy through genome sequencing, targeted metabolomics, and machine learning
Source: BMC Med Genomics. 2025 Nov 19;18:187. doi: 10.1186/s12920-025-02261-x (PMC12628566; doi:10.1186/s12920-025-02261-x)
Supplement: Supplementary file 1 — Supplementary Material 1. [file 12920_2025_2261_MOESM1_ESM.docx]

**XieY et al. 2025. Improving newborn screening accuracy through genome sequencing, targeted metabolomics, and machine learning.**

**Supplementary Figure 1A**


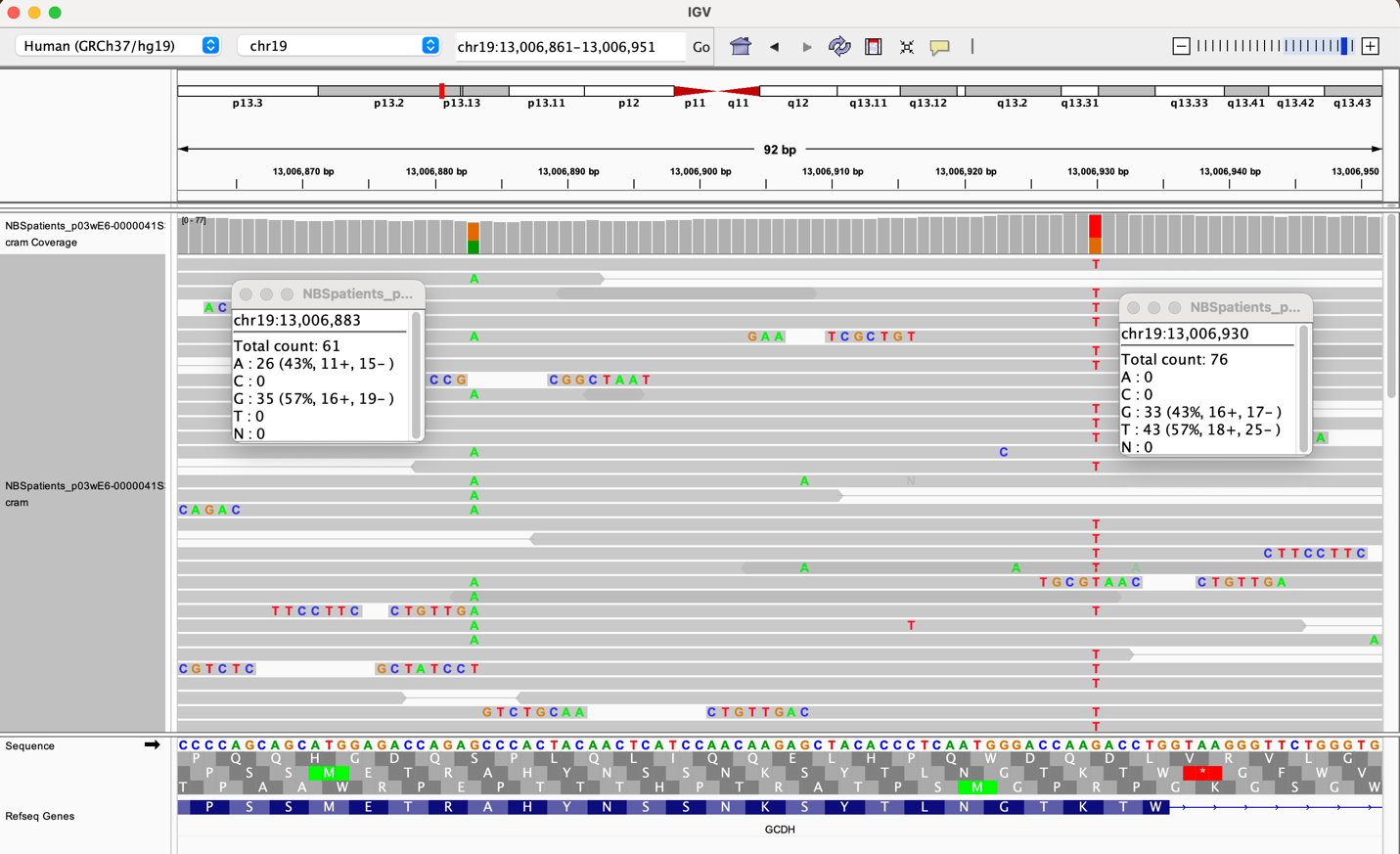


Sample: NBSpatients_p03wE6-0000041S3231

Variant 1: 19-13006883-G-A

GCDH:NM_000159.4:exon7:c.G583A:p.Ala195Thr(het)

Variant 2: 19-13006930-G-T

GCDH:NM_000159.4:exon7:c.G630T:p.Lys210Asn(het)

**Supplementary Figure 1B**


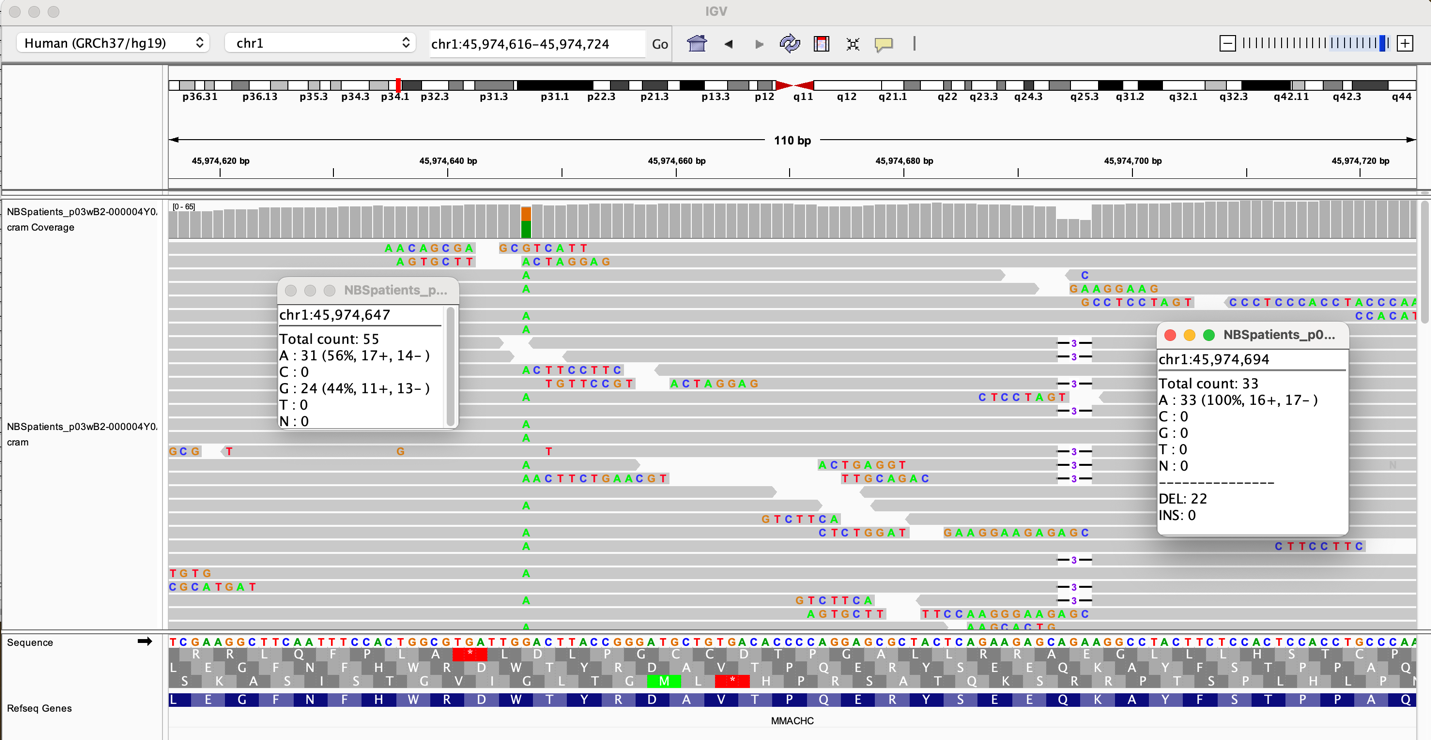


Sample: NBSpatients_p03wB2-000004Y0A053

Variant 1: 1-45974647-G-A

MMACHC:NM_015506.3:exon4:c.G609A:p.Trp203Ter(het)

Variant 2: 1-45974693-CAGA-C

MMACHC:NM_015506.3:exon4:c.658_660del:p.Lys220del(het)

**Supplementary Figure 1C**


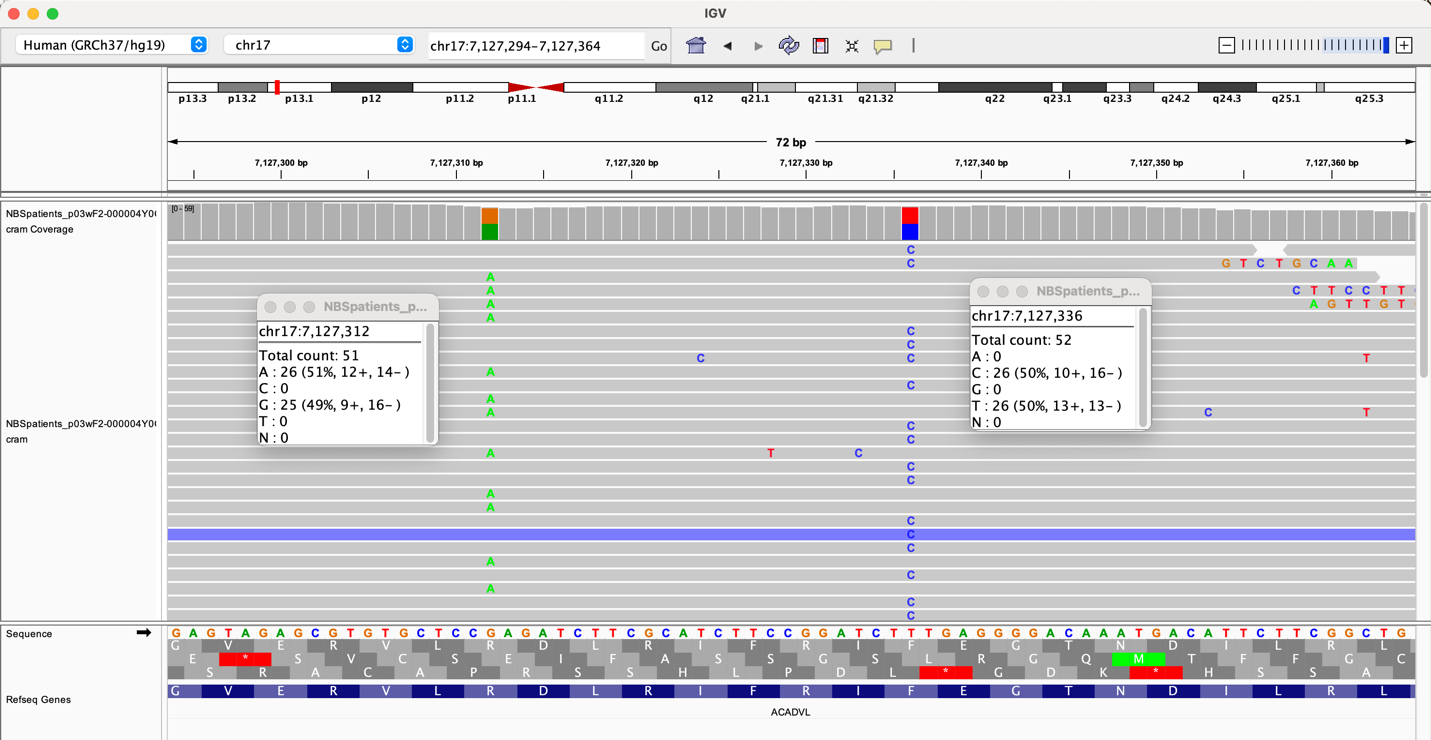


Sample: NBSpatients_p03wF2-000004Y0G077

Variant 1: 17-7127312-G-A

ACADVL:NM_000018.4:exon14:c.G1358A:p.Arg453Gln(het)

Variant 2: 17-7127336-T-C

ACADVL:NM_000018.4:exon14:c.T1382C:p.Phe461Ser(het)

**Supplementary Figure 2**


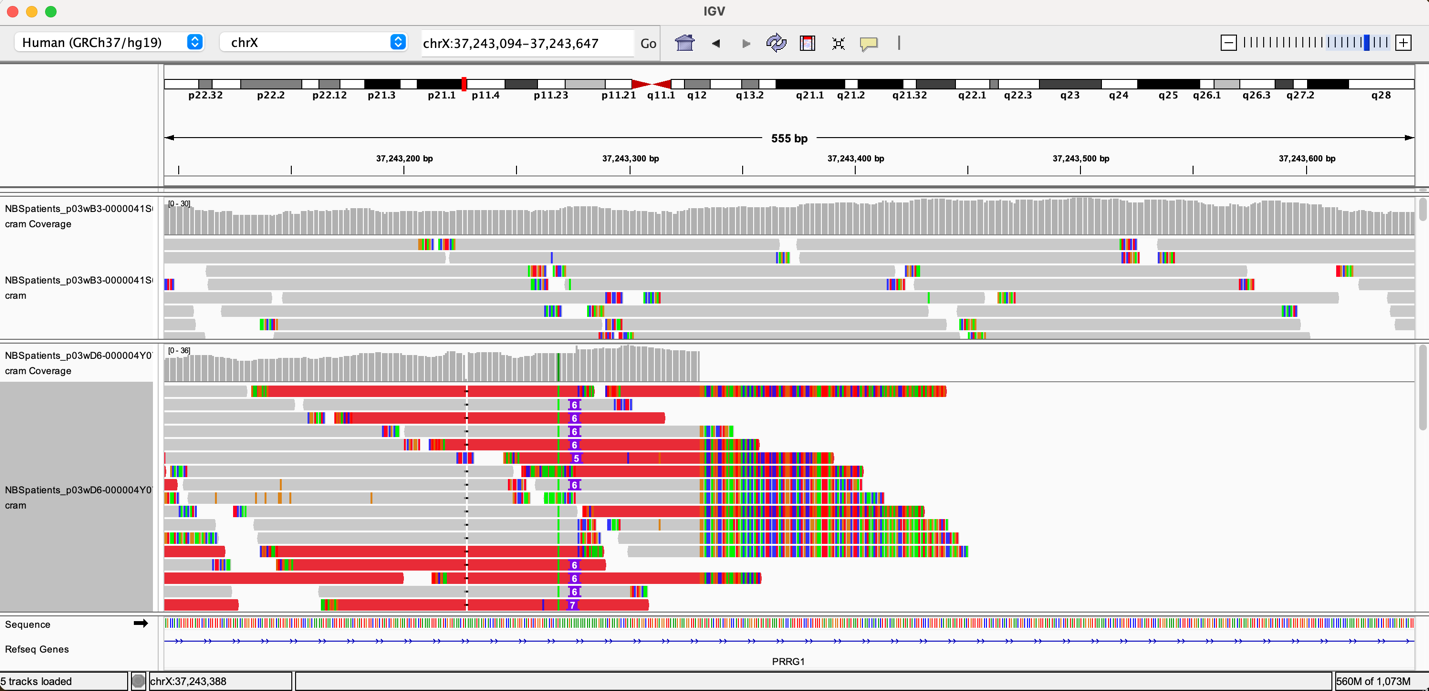

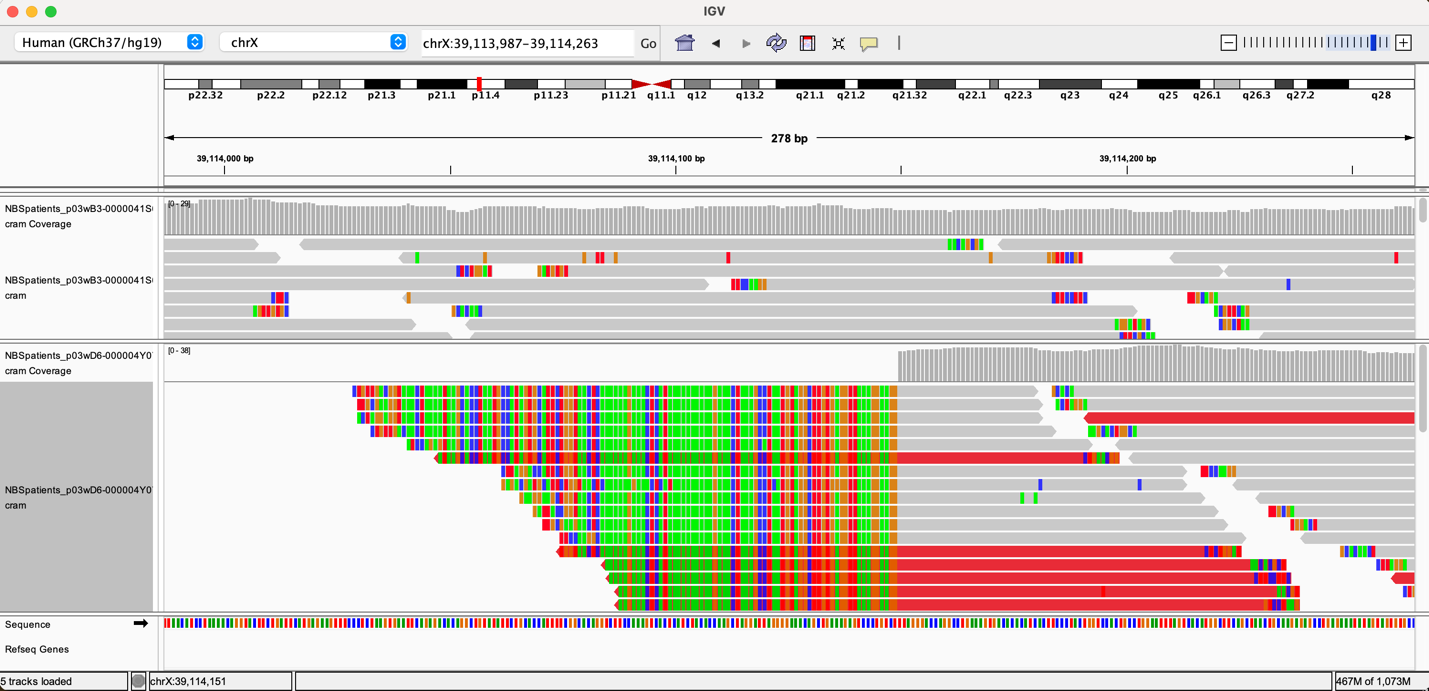


Sample: NBSpatients_p03wD6-000004Y07023

Variant 1: A 1,870,820bp likely pathogenic deletion of chromosome Xp11.4 involving 12 genes, including the OTC gene (PRRG1, LANCL3, XK, CYBB, H2AP, SYTL5, SPRX, RPGR, OTC, TSPAN7, MID1, LINC01281). Minimal interval chrX:37,243,330-39,114,150x0, GRCh37).
